# Supplementary material for: Are carbon emissions trading and green financial instruments synergistic? -Comprehensive quantitative research based on content analysis
Source: PLoS One. 2024 Mar 7;19(3):e0298601. doi: 10.1371/journal.pone.0298601 (PMC10919601; doi:10.1371/journal.pone.0298601)
Supplement: S1 Data — (ZIP) [file pone.0298601.s001.zip › Data/地级市Z_F工作报告.zip!/░▓╗╒/│╪╓▌╩╨/└┤╫╘╣1⁄2╓┌║┼í╛┬φ┐╦╩2╛▌═°í┐_2016.docx]

各位代表：

　　现在，我代表市人民政府，向大会报告工作，请予审议，并请市政协委员和其他列席人员提出意见。

**一、2015年工作和“十二五”发展回顾**

　　过去的一年，全市人民在市委的坚强领导下，深入贯彻落实党的十八大、十八届三中、四中、五中全会和习近平总书记系列重要讲话精神，主动适应经济发展新常态，积极应对经济下行压力，综合施策，精准用力，协调推进，经济社会发展总体平稳，稳中有进、稳中有好，较好地完成了年初确定的各项目标任务。初步测算，全市生产总值550亿元、增长8.5%左右，财政收入95.8亿元、增长4%，固定资产投资600亿元、增长11.5%，社会消费品零售总额181亿元、增长12%；进出口总额5.1亿美元、增长24%；城乡居民人均可支配收入分别增长9.4%、9.5%；节能减排完成省控目标。

　　一是着力稳增长，实体经济保持平稳运行。认真落实省、市促进经济持续健康发展各项政策措施，建立市政府负责同志精准帮扶重点企业和联系重点项目制度，加强一线调度，狠抓政策落地，解决矛盾问题，开工建设红太阳新材料一期等28个亿元以上工业项目，超元半导体等30个项目建成投产，全年完成工业投资345亿元、增长14%，新增规模工业企业84家，规模工业增加值178亿元、增长10.6%；20个亿元以上文化旅游项目完成投资30.4亿元，杏花村文化旅游区民俗体验区等4个景区建成开放，全市接待国内外游客4637万人次，旅游总收入478亿元，分别增长12.2%、13.3%。转变支持实体经济方式，整合市本级各类专项资金，设立产业发展基金，兑现工业、旅游、商贸、农业等产业扶持政策1.1亿元。加大清费减负力度，取消、免征、暂停、降标行政事业性收费、政府性基金项目64项，结构性减税和普遍降费政策减轻企业负担2.8亿元。强化要素支撑，清理盘活闲置低效土地1.04万亩，“工学一体”就业就学试点协议招生（工）1300人，充实国有融资性担保机构资本金2.93亿元，建立“4321”政银担风险分担和补偿合作机制，帮助231户企业实现信贷融资9.3亿元，28家企业通过并购重组、二次招商盘活资产20多亿元，九华股份在主板首发上市，17家企业在“新三板”、“四板”挂牌，3家企业吸引风投资金1亿元，全年社会融资总量130亿元，其中中小微企业新增贷款12亿元、增长5.4%。突出刚性需求，出台促进房地产业健康转型发展的政策，推行棚户区改造“房票”、货币化安置改革，房地产市场保持健康平稳。

　　二是着力调结构，产业转型升级步伐加快。出台“调转促”实施方案，修订促进工业转型升级等政策措施，制定电子信息产业发展规划及政策，新引进落户电子信息产业项目25个，竣工投产14个；完成技改投资225亿元，增长37%，建成21个“两化融合”项目；设立1亿元创业天使投资基金，新增高新技术企业12家、省级创新型（试点）企业10家，组建省级企业研发机构11个，发明专利授权量增长124%，全年战略性新兴产业产值87亿元、增长28%，高新技术产业产值285亿元、增长23%。细化落实皖南国际文化旅游示范区建设50项重点任务，新增中国特色景观旅游名镇名村1个、省级特色景观旅游名镇名村9个，成功举办第五届绿运会等4项全国性赛事和综合性展会，与黄山等市建立旅游联合发展机制，开展市域旅游整体营销73场（次），众筹、物联网、互联网+等商业模式创新不断涌现。加快发展新型服务业态，全国唯一的国家森林生态标志产品电商交易平台落户池州，开启现代服务业转型发展和“互联网+林业”的崭新模式；电商孵化创业园实现县区全覆盖，引进落户大型电商龙头企业8家，集聚电商创业实体近300户，限上批发零售业网上商品零售额增长1.1倍；发展各类养老机构79家；新增限上商贸流通企业52家。大力发展现代农业，全年粮食总产突破70万吨，农业增加值75亿元、增长4%，规模农产品加工企业产值108亿元、增长13.5%；建立31个粮棉油高产创建示范片，新建和改造无性系良种茶园10854亩，建设蔬菜基地1000亩，新增“三品一标”认证11个，池州国家农业科技园区获科技部批准；新增土地流转8.04万亩，组建农业产业化联合体12个，发展家庭农场540个、农民专业合作社179个，建设高标准农田23.8万亩。

　　三是着力抓统筹，城乡环境面貌日益改善。《城市总体规划（2013-2030年）》获批实施。成功争取全国首批海绵城市建设试点，5个首批示范项目和8个设计变更项目全面开工，40个社会投资项目全面落实建设技术要求，试点做法被国办信息专题刊发。扎实推进文明创建“十大集中专项行动”，启动实施主城区物业管理“三年提升行动”，完成 3个老旧小区环境整治，一批不文明的顽症明显改观，荣获全国文明城市提名城市。县城规划建设管理“六项行动”初显成效，池州在全省督查考评中位居第3，三县在皖南片评分中名列第一方阵。整合和调动社会资金投入3.6亿元，实施美好乡村建设项目1035个，改造农村危房8800户。宁安高铁、东九高速、望东长江公路大桥南岸接线建成通车，池州长江公路大桥建设加快。新改建城市道路、管网管线、城市绿道156公里，开工城市生活垃圾无害化处理项目，建成数字化城市管理平台。完成国省干线公路建设及大中修122公里、县乡公路升级改造及村级公路网化工程254公里，加固改造农村危桥101座。解决14.8万农村人口饮水安全问题，完成各类水利建设投资5.3亿元。农网改造升级工程治理低电压6831户。建成全国生态乡镇31个、生态村7个，省级森林城镇5个、森林村庄52个。实施232个大气污染防治项目，空气质量优良率94.2%，PM10平均浓度55微克/立方米。人工造林7.6万亩，治理水土流失20平方公里。整顿关闭非煤矿山13家，“三线三边”矿山生态恢复治理17家。升金湖成为全省首个国际重要湿地自然保护区。

　　四是着力促改革，创新创业活力充分激发。全面实施政府权力、责任、涉企收费“三项清单”制度，清理减少市级行政审批项目25个，全面取消市直部门非行政许可审批事项。深入推进商事制度改革，全年净增私营企业1949户、个体工商户6730户。调整完善市公共资源交易机构职责，池州列入国家电子招标投标交易平台试点城市。城市污水处理及市政排水设施PPP项目成为全国同类第一个签约运营的示范项目和财政部7个经典案例之一，创造了政府与社会资本合作的“池州模式”。整合站前区、齐山-平天湖风景区，设立平天湖风景区管委会。完成市卫生和计生、工商和质监、港口和地方海事、不动产统一登记职能整合。全面实现“一元化”户口登记模式。公务用车制度改革方案获批实施。加强与央企、知名民企、上市公司、商会协会的对接合作，全年引进规模项目470个、建成投产300个，省级以上园区引进5亿元以上大项目21个，其中10亿元以上5个，开工建设5个，亿元以上省外项目到位资金320亿元、增长7.8%，实际利用外资3.3亿美元、增长10%左右。

　　五是着力惠民生，人民群众福祉不断增进。全年民生类支出121.1亿元、增长10.5%，占财政总支出的84.5%，其中省33项民生工程和市10件惠民实事投入资金28.7亿元。创业就业和新型职业农民培训1.02万人，城镇新增就业1.91万人，城镇登记失业率3.4%。企业退休人员养老金人均月增192元，农村五保供养补助标准人均年增1000元，“新农合”人均筹资标准提高到480元，城镇居民大病保险实现即时结算。建立城乡低保动态管理、孤儿基本生活保障机制，清退不符合标准的低保对象3588人，符合条件孤儿全部纳入保障范围。市老年公寓二期工程主体完工，新增社会养老床位300张、农村敬老院床位200张，建成农村幸福院51个。保障性住房开工13305套、基本建成13075套、新分配入住9998套，山区库区农村住房保险试点参保35.4万户。启动10个贫困村、400户贫困户光伏电站建设，全市减少贫困人口1.53万人。率先通过国家义务教育发展基本均衡县（区）评估认定，全面完成义务教育阶段学校标准化建设，实施188所义务教育薄弱学校办学条件改善项目，新改扩建幼儿园8所。基本公共卫生服务人均补助标准提高到40元，市第三人民医院建成试运营。新增5个中国传统村落、2个全国生态文化村和6个全省首批“千年古镇”、“千年古村落”，完成10个农民文化乐园试点、315个广播电视村村通建设。华龙洞古人类遗址考古发掘工作取得重大进展。10人入选“中国好人榜”、“安徽好人榜”。人口自然增长率3.6‰。妇女、儿童、老年人和残疾人权益得到保障。完成第二次全国地名普查年度任务，供销、气象、地震、民族、宗教、对台、外事、侨务、档案、保密、地方志、新闻出版、社会科学等各项工作取得新成效。第三届社区居委会换届选举顺利完成。“六五”普法通过验收，信访秩序规范有序，社会治安防控体系不断完善。安全生产形势持续稳定，食品药品安全、市场供应和价格监管积极有效，应急管理不断加强。市光荣院、革命烈士陵园纪念馆完工，国家安全、国防动员、人民防空和民兵预备役等工作扎实有效。

　一年来，我们以开展“三严三实”专题教育为抓手，全面加强政府自身建设。深入推进依法行政，健全政府学法制度、重要文件学习制度、政府法律顾问制度，坚持科学民主依法决策，健全决策程序，依法依规解决复杂疑难问题。自觉接受人大法律监督和工作监督，主动接受政协民主监督、人民群众监督和新闻舆论监督，认真听取各民主党派、工商联、无党派人士和各人民团体的意见，办理人大代表议案和建议168件、政协委员提案342件，办结率均为100%。深化政务公开和政府信息公开，建立新闻发布和政策解读制度，回复办理市长信箱、市长热线和网民留言6642条。加强政府预算管理，实行预算公开，厉行勤俭节约，“三公”经费下降12%。完善政府债务管理制度，有效防范政府债务风险。认真落实中央八项规定和省委、市委规定要求，严格落实政府系统党风廉政建设主体责任，建立重大决策和重要工作落实问责制度，推进审计监督“六个全覆盖”，扎实开展发生在群众身边的腐败问题专项治理，自身建设取得新成效。

　　各位代表！过去的一年是“十二五”收官之年，成绩来之不易。回首“十二五”，在省委、省政府和市委的坚强领导下，我们团结拼搏，砥砺奋进，成功战胜多重自然灾害的重大挑战，有效应对“三期叠加”形势下宏观环境复杂多变的严峻考验，较好地完成了“十二五”规划确定的主要任务，经济社会发展取得了令人鼓舞的巨大成就。

　　——这五年，是我们深入推进皖江城市带承接产业转移示范区建设，经济实力跨上新的台阶、转型发展取得重大突破的五年。地区生产总值连续跨上2个百亿元台阶，年均增长10.6%，人均生产总值超过6000美元；财政收入实现翻番，年均增长17.2%；规模工业增加值年均增长16.1%，连续5年超过全省平均水平，亿元以上工业企业户数是“十一五”末的3.2倍，工业化率比“十一五”末提高1.7个百分点。江南产业集中区落户我市，市开发区晋升国家级开发区，贵池工业园获批省级高新区，4个园区产值突破百亿元。“两高一首”产业定位更加清晰，电子信息产业从无到有，高性能芯片填补省内空白，我市全省集成电路产业副中心地位更加突出。研究与试验发展经费投入比“十一五”末增长5倍，专利授权量年均增长40%，高新技术产业产值年均增长40%，战略性新兴产业产值年均增长16.5%。三次产业结构由“十一五”末的15.2∶46.6∶38.2调整为13.2∶46.1∶40.7。

　　——这五年，是我们扎实推进皖南国际文化旅游示范区建设，旅游支撑地位大幅提升、特色发展取得重大成效的五年。列入皖南国际文化旅游示范区核心区，大愿文化园等一批标志性工程建成开放，杏花村文化旅游区等一批战略性项目加快建设，新增4A级景区9个，市山县区旅游联动发展初显成效，乡村旅游蓬勃兴起，游轮旅游、航空旅游等高端旅游消费日益升温，接待国内外游客、实现旅游收入分别是“十一五”的3.6倍、4.3倍。旅游与文化、生态、体育、会展、农业融合发展取得突破，电子商务、现代物流、会展经济、体育产业、总部经济等新兴业态快速成长，社会消费品零售总额年均增长14.7%、稳居全省前列。池州荣获中国十大特色休闲城市、携程年度最具潜力旅游目的地称号。

　　——这五年，是我们创新推进全国生态文明示范区建设，美丽池州成为重要品牌、统筹发展取得重大进展的五年。中心城市加速由外延式扩张向内涵式提升转变，跻身全国低碳城市试点市、国家森林城市、中国人居环境奖城市、全国绿色生态示范城区、全国可再生能源建筑应用示范城市、全国生态保护与建设示范区和安徽省生态市、水生态文明建设试点市行列，生态宜居的城市特质进一步彰显。县城规划建设管理水平不断提高，三县全部通过国家生态县技术评估，建成59个省级美好乡村示范村。单位生产总值能耗比“十一五”末下降15%以上，化学需氧量、氨氮、二氧化硫和氮氧化物等四项主要污染物减排完成省定目标，大气环境质量稳居全省前两位。

　　——这五年，是我们大力推进对外开放和招商引资，有效投入持续扩大、经济发展后劲显著增强的五年。完成固定资产投资2265亿元，年均增长22%，其中工业投资1252亿元，年均增长25%。招商引进亿元以上项目336个，其中10亿元以上14个，建成投产117个，11家央企、上市公司落户我市。九华山机场等一批重大交通项目建成运营，池州跨入“航空时代”、“高铁时代”，“水、陆、空”立体化交通体系全面形成。国道总里程增加330公里，万人公路密度居全省第2，港口吞吐能力和吞吐量分别是“十一五”末的1.69倍、1.57倍。水库除险加固恢复兴利库容7000万立方米，城市排涝标准提高到20年一遇。进出口总额19.3亿美元，实际利用外资12.9亿美元，分别是“十一五”的3倍、2.4倍，池州荣获全国外贸百强城市称号。

　　——这五年，是我们全面推进改革创新和法治建设，政府施政行为明显规范、市场主体活力充分迸发的五年。政府机构改革、商事制度改革、农村综合改革等各项改革扎实推进，率先在全省开展风景区相对集中行使行政处罚权、自然保护区联合执法改革。深化行政审批制度改革，加强事中事后监管，乡镇为民服务中心和村级代理工作站基本实现全覆盖，成为全省行政审批事项最少、发展环境最优的城市之一。国有资产、财政性资金、政府性债务实现全口径管理，公共资源配置、政府性投资项目建设、公共资源交易逐步全过程规范，清理盘活国有存量资产30亿元、财政存量资金12.5亿元。新增6家银行业金融机构，企业主板上市实现“零的突破”，直接融资比重提高到20%。私营企业、个体工商户户数分别是“十一五”末的2.1倍、1.3倍，民营经济增加值比重比“十一五”末提高3.2个百分点。

　　——这五年，是我们着力推进民生实事和社会建设，基层基础日益夯实、和谐稳定大局持续巩固的五年。城乡居民收入年均分别增长11.5%和13.5%。民生工程和惠民实事完成投入131.9亿元，解决一批群众多年想办、急需办的民生设施、社会保障问题。新增城镇就业8.62万人，城乡基本养老保险体系基本形成，社会救助制度全面建立，“一老一小”保障水平不断提高，住房保障能力明显增强，城乡低保、农村五保标准实现翻番。教育、文化、卫生等基本公共服务体系进一步健全，义务教育实现基本均衡，高等院校全日制在校生规模实现翻番，基本药物制度全面建立，建成开放市博物馆、图书馆、科技馆、文化馆、青少年活动中心，新增国家级、省级文物保护单位22处。主城区标准化社区实现全覆盖，群众对民生工程满意率和对社会治安的安全感多年居全省前列，实现全国双拥模范城“三连冠”，荣获全国信访工作先进集体称号。

　　各位代表!五年的奋斗充满艰辛，取得的成就令人振奋，这是省委、省政府和市委坚强领导的结果，是市人大、市政协监督支持的结果，是全市人民同心同德、顽强拼搏、主动作为的结果。在此，我代表市人民政府，向广大工人、农民、知识分子、干部、离退休老同志，向驻池解放军指战员、武警官兵和政法公安干警，向各民主党派、各人民团体和各界人士，向关心、支持池州发展的中央和省驻池单位，向在我市创业的投资者、建设者，向给予市政府高度信任和倾力支持的各位人大代表、政协委员，表示衷心的感谢！

　　各位代表!回顾五年来的奋进历程，我们更加深刻地体会到，要把池州各项事业不断推向前进，必须始终坚持既定的发展战略，在遵循发展规律中与时俱进创新思路，在吃透中央精神中紧密结合池州实际，保持定力，持久用力，一任接着一任干，一张蓝图画到底；必须始终坚持发挥池州的特色优势，坚定不移发展与国家产业政策同向、与池州生态环境相融的产业，夯实城市发展支撑，增强城市经济实力，带动广大群众创业就业，共建共享改革发展成果；必须始终坚持发挥好政府与市场“两个作用”，政府重在“放、管、服”同步用力，创造公平公正公开的发展环境，让各类市场主体创新创业创造活力在池州大地上充分迸发；必须始终坚持加强能力建设和作风建设，强化学习研究，强化使命担当，强化制度建设，严守纪律和规矩，按照市委的部署，充分调动各方面的积极性，团结协作，苦干实干，一着不让抓落实。这些经验体会，要在“十三五”工作中继续发扬深化。

　　在肯定成绩的同时，我们也倍加清醒地看到，在前进道路上还面临不少困难和问题。主要是：发展不足，发展不优，经济增速换挡、结构调整阵痛、动能转换困难相互交织，有效需求乏力和有效供给不足并存，创新能力不足，调结构转方式促升级任务艰巨；经济回稳动力和下行压力仍在相持，市场需求不足，企业融资、配套、用工等综合成本居高不下，经济增长基础还不稳固，规模工业增加值、财政收入等少数指标实际完成情况与年初确定的计划有缺口；基本公共服务供给能力和质量还不高，居民收入持续增长难度加大；政府职能转变还不到位，依法行政水平需要进一步提升，发展环境需要进一步优化，解放思想、开放合作的力度需要进一步加大，少数干部精神不振、作风不实和能力不足问题交织，党风廉政建设需要进一步加强。对此，我们要以对人民、对历史高度负责的态度，认真加以解决。

**二、“十三五”时期的奋斗目标和任务**

　　根据市委《关于制定池州市国民经济和社会发展第十三个五年规划的建议》，围绕“人均争先、生态领先、环境居先、文明创先、民生优先”，综合考虑，今后五年经济社会发展的主要目标是：

　　——经济保持中高速增长。地区生产总值年均增长8.5%左右，达到850亿元，努力向千亿级冲刺，人均地区生产总值9300美元，力争达到1万美元，主要经济总量指标和人均主要经济指标在全省位次进一步提升。

　　——调结构转方式促升级取得实质性进展。三次产业结构和产业内部结构显著优化，产业迈向中高端水平，高新技术产业增加值占规模工业比重达到48%，现代服务业增加值占服务业比重达到50%，农产品加工产值与农业总产值比例达到1.5，三次产业比例优化至11:47.5:41.5。

　　——绿色发展成效显著。生态文明重大制度基本确立，生产方式和生活方式绿色、低碳水平不断上升，能源和水资源消耗、建设用地、碳排放总量和强度得到有效控制，大气、水、土壤等污染得到有效整治，主要污染物达标排放。

　　——社会文明程度大幅提升。中国梦与社会主义核心价值观更加深入人心，和美文化广泛弘扬，公民思想道德素质、科学文化素质、健康素质明显提高，全社会法治意识不断增强，建成全国文明城市。

　　——人民生活水平和质量显著提高。常住居民人均可支配收入力争达到全国平均水平，就业、教育、文化、社保、医疗、住房等公共服务体系更加健全，基本公共服务均等化水平稳步提高，现行标准下农村贫困人口实现脱贫，贫困地区整体脱贫。

　　——各方面制度更加健全。重点领域和关键环节改革取得决定性成果，法治政府基本建成，司法公信力明显提高，市场在资源配置中的决定性作用更加突出，政府作用更好发挥，形成一批在全省乃至全国有影响力的改革成果。

　　重点推进五项战略任务：

　　（一）坚持创新发展，构建现代产业新体系。紧紧依托“三区一带”战略平台，推动产业向中高端发展，到2020年，建成1-2个省级战略性新兴产业集聚发展基地，力争工业增加值350亿元，规模企业1000户，亿元以上企业300户，10亿元以上企业30户，5家企业IPO上市。加快旅游全域化发展，到2020年，力争打造2个具有国际水准的精品景区、2个具有国际竞争力的特色旅游产品、3个国家级示范基地和度假区、3条融入区域大循环的精品线路、3家主营收入5亿元以上的龙头企业，建成国家全域旅游示范市和全国旅游标准化示范市。做强大健康产业，到2020年，建成10个以上以“养”为主体、“健”为支撑、“食”为特色、“医”为保障的健康养老养生产业示范基地，打造国内一流、国际知名的健康池州，争创国家现代服务业集聚区。大力推进农业现代化，加快构建现代农业产业、生产、经营“三大体系”，提高农业综合效益和竞争力。坚持创新驱动，到2020年，新增高新技术企业50家以上，高新技术产业产值、规模以上工业新产品产值实现翻番。

　　（二）坚持协调发展，形成城乡统筹新格局。促进区域协调发展，支持贵池区建设皖江创新强区、皖南旅游集散中心，东至县建设新型化工制造强县、绿色农产品基地，石台县建设中国原生态最美山乡，青阳县建设机电装备制造强县、环九华山休闲养生度假基地，江南产业集中区建设现代化滨江产业新城，九华山建设世界佛教文化观光胜地，市开发区建设国家低碳工业示范园区，平天湖风景区建设现代服务业集聚区、国际生态休闲度假区。全面提升城乡基础设施承载力，着力构建外部畅通和内部循环的综合交通体系、普惠泛在安全的信息化支撑体系、高效安全的现代能源体系、现代化的水利保障体系。

　　（三）坚持绿色发展，提升美丽池州新形象。完善主体功能区布局，制定实施市县（区）主体功能区规划，到2020年，国土空间开发强度控制在15%以内。加大环境治理与生态保护力度，到2020年，森林覆盖率达到60%，城市建成区绿化覆盖率达到45%，大气环境质量优良率稳定在95%以上，耕地土壤环境质量达标率不低于82%，矿山复绿率不低于90%。推进资源节约及高效循环利用，到2020年，万元工业增加值用水量下降25%，单位生产总值建设用地消耗下降10%，创建2个国家循环化改造试点园区。

　　（四）坚持开放发展，构建开放型经济新体制。全面融入国家开放总体布局，积极投入长江经济带建设，扩大与“一带一路”沿线地区的经贸合作和文化交流，广泛参与长三角城市群建设，推进安池铜城市组群、省际毗邻城市组团发展，主动对接京津冀协同发展。打造高水平开放平台，力争九华山机场申建国家一类航空运输口岸、开通国际（跨境）航线，扩大池州港口岸对外籍船舶开放，争取设立保税物流园区等海关特殊监管区域，到2020年，建成4个产值超两百亿元园区，进出口总额达到8亿美元。提升承接产业转移水平，“十三五”期间，力争引进亿元以上项目660个，到位资金1900亿元。深化重点领域和关键环节改革，加快社会信用体系建设，着力优化政务环境、商务环境、人文环境、法治环境，激发创新创业创造活力，到2020年，民营经济增加值比重达到65%。

　　（五）坚持共享发展，实现人民福祉新提升。坚决打赢脱贫攻坚战，确保如期完成脱贫任务，到2020年，所有贫困人口与全市人民同步进入全面小康社会。全面发展社会事业，到2020年，全面普及15年基础教育，新增劳动力普遍接受12年以上教育，高等教育毛入学率达40%以上，每万人口执业（助理）医师达到2.2人，每千名老人拥有养老床位达到45张。加强就业创业，建立面向人人的创业服务平台和创业孵化示范基地，推行终身职业技能培训制度，努力实现充分就业，推动实现更高质量创业。健全多层次社会保障体系，实施全员参保计划，基本实现法定人员全覆盖，全面建成公平统一的城乡居民养老保险制度，基本建立全市统一的城乡居民基本医疗保险制度。夯实社会治理基层基础，加快法治池州建设，完善社会治安防控体系，确保社会和谐稳定。

　　实现上述目标任务，我市“五位一体”发展必将取得重大进展，一个经济繁荣、环境优美、社会和谐、生活富裕的新池州，必将屹立在长江之滨，展现出更加广阔的光明前景！

**三、2016年的主要工作**

　　今年是全面实施“十三五”规划的第一年，做好今年的工作意义十分重大。综合考虑，今年经济社会发展的主要目标是：全市生产总值增长8.5%，财政收入与经济增长同步，规模工业增加值增长10%，固定资产投资增长10%，社会消费品零售总额增长10%，进出口总额增长8%，接待国内外游客、旅游收入分别增长10%、12%，城乡居民人均可支配收入分别增长8.5%、9%，城镇登记失业率控制在4.5%以内，居民消费价格涨幅控制在3%左右，节能减排达到省控目标。

　　重点抓好以下八个方面工作：

　　（一）加快产业转型升级，夯实城市经济支撑

　　加快工业经济转型发展。完善电子信息产业发展规划，组建产业发展专家咨询委员会，支持池州高新区与合工大组建微电子研究院，推动江南产业集中区、市开发区、池州高新区协同错位招商，力争在引进龙头大项目和产业链关键环节项目上取得突破，争取列入省级电子信息产业集聚发展基地。支持省级以上园区争创省级战略性新兴产业集聚发展基地，完善生产性服务业配套功能，加强招商引资和对外合作，提升园区企业投资强度和税收贡献，全市园区工业总产值增长12%、税收增长8%以上。实施60个亿元以上重点工业项目，确保30个项目开工建设，30个项目建成投产，继续加快海螺四期、九华发电二期等重大项目前期工作。建设100个以上“两化融合”等技术改造项目，完成技改投资150亿元。完善产业发展基金、创业天使投资基金监管运行机制，以股权投资形式支持一批种子期、初创期、早中期高成长性企业成长裂变，新认定和培育高新技术企业、省级创新型（试点）企业15家以上，高新技术产业产值增幅超过全省平均水平，战略性新兴产业产值占规模工业的比重提高1个百分点。

　　大力发展现代生态农业。实施粮棉油高产创建和绿色增长模式攻关行动，建设高标准农田12.4万亩，创建绿色增产模式示范点20个，确保粮食产量稳定在66万吨以上。发挥国家森林城市品牌优势，依托国家森林生态标志产品电商交易平台，大力发展生态农业和森林经济，新改建8000亩无性系良种茶园，建设九华黄精、西山焦枣等6大森林生态产品基地，新增“三品一标”认证10个以上，积极开发生态渔村、山水人家、采摘篱园等乡村旅游产品，加大龙头企业招商力度，引导发展农产品深加工，实施互联网+，促进一二三产业融合发展。积极创建省级现代生态农业产业化示范市，加快池州国家农业科技园区和4个省级现代农业示范区建设，争创省级以上家庭农场10家、农民专业合作社示范社20个，组建产业化联合体10个。推广农业物联网等新技术，强化基层农技推广服务、重大动植物疫病防控和农产品质量安全监管，扩大农业保险覆盖面。完成整市推进农村土地承包经营权确权登记颁证试点工作，稳步推进集体产权制度试点村、国有林场、小型水利工程建管体制、集体林权制度配套等各项改革。

　　提升服务业发展水平。认真落实国家和省加快服务业发展的政策措施，积极培育新的消费热点，服务业增加值增长8%以上。全面开展富硒资源普查工作，制定大健康产业发展规划和政策，大力发展健康养老养生、体育休闲、文旅体验等产业，争创国家医养结合试点示范基地。大力发展电子商务，新增3家以上千万元销售额电商企业，积极创建国家电子商务示范基地，促进跨境电商、农村电商、快递业有序发展。加快发展现代物流，建成运营前江仓储物流园等5个重大物流项目。大力发展金融服务业，加快艾可蓝IPO首发上市，新增6家以上企业在“新三板”、“四板”挂牌，全市直接融资30亿元以上；大力引进域外银行、证券、基金公司等金融机构，积极运用企业债、中期票据、融资租赁、资产证券化等市场化融资方式，规范发展互联网金融、小贷公司等类金融机构，打击非法集资，加强金融市场监管，切实防范金融风险。

　　支持实体经济发展。继续实施市政府负责同志精准联系帮扶重点企业制度，落实结构性减税、普遍性降费政策，清理各种不合理收费特别是垄断性中介服务收费，多措并举减轻企业负担。健全银企对接、续贷过桥、"4321"政银担风险分担和补偿合作机制，推广“税融通”贷款业务，确保小微企业贷款“三个不低于”和涉农贷款增速高于全部贷款增速，全年社会融资总量130亿元，其中银行新增信贷投放30亿元以上，有效解决中小企业融资难。落实户籍制度改革措施，建立租购并举的住房制度，鼓励社会资本购买库存商品房，取消过时的限制性措施，扩大有效需求，积极化解房地产库存，促进房地产市场健康转型发展。

（二）推进旅游全域发展，提升旅游综合竞争力

　　高起点高水平编制规划。坚持以国际化为引领，围绕创建国家全域旅游示范市，组建旅游文化产业发展专家咨询委员会，招募一流旅游规划团队，编制全市旅游产业发展规划、全市乡村旅游发展规划，构建层次清晰、承接有序的规划和管理体系，制定分年度的规划实施方案，严格按规划组织实施。

　　高端策划开展对外宣传营销。市山县区整体联动，高起点策划大型营销推介活动，在央视及境内外重要客源地城市媒体常态化推广池州旅游主题形象，组织参加全省"1+N"旅游宣传推广活动，积极开展“池州九华山号”高铁列车宣传营销，完成九华山机场航空口岸临时开放申报工作，继续加强机场、游轮航线城市促销，办好绿运会、佛文化用品博览会、杏花村文化旅游节等一批在全国有影响力的赛事展会，全面提升池州旅游品牌形象。

　　推进旅游重点项目建设。坚持以项目为抓手，实施“旅游+产业专项行动计划”，推动旅游与文化、生态、体育、农林、商贸、互联网深度融合发展，重点加快杏花村文化旅游区等20个亿元以上文化旅游项目建设，确保仙寓山富硒养生度假区等5个项目开工建设，3个项目建成运营，力争大愿文化园成功创建国家文化产业示范基地，新增4A级景区1家、省优秀旅游乡镇5个、旅游示范村5个、4星级以上农家乐8家，开发休闲度假、乡村回归等10类以上个性化、参与式旅游产品，培育10 个以上有特色、有影响力的“游客乐购旅游商品”，评选建设百佳摄影点、观赏地。

　　完善旅游公共服务体系。启动市域旅游环线和主城区、县城旅游集散中心规划建设，建成运营池州港新旅游口岸，实施3A级以上景区“一票通”和旅游大巴通达工程，全面完成高速公路、国省道及主城区通往4A级以上景区道路沿线旅游标识牌建设，新建和改造50座旅游厕所，力争4A级以上景区智慧旅游建设全覆盖。

　　创新旅游发展体制机制。坚持深化旅游改革，努力在区域合作机制、促进产业融合发展、要素资源配置等方面先行先试，办好示范区第二次联席会议，加强与示范区城市的合作，建立市山县区旅游联动发展协调调度制度，推进项目互补、营销互动、客源互送。健全平天湖风景区管理体制机制，组建旅游开发公司，力争引进1家大型旅游企业，整体策划、联动发展以平天湖、杏花村、九华天池等景区为核心的主城区旅游。完善大愿文化园管理运营机制，加大升金湖联合执法力度。深化旅游市场环境整治行动，加强旅游人才引进和培训，提升旅游服务水平。认真实施职工带薪休假制度，促进旅游休闲消费。

　　（三）提升城乡基础功能，加快统筹发展步伐

　　加快重大交通项目建设。建成望东长江公路大桥，加快池州长江公路大桥和高岭-牛头山、东流-尧渡、木镇-青阳等3条国省道一级公路以及江口、梅龙、前江、香隅等4大港区建设，继续推进九华山机场扩建、六安-景德镇铁路池州段、黄冈-安庆-九华山-黄山城际铁路、宣城-池州-宿松城际铁路、武汉-池州-杭州客运专线、池州-石台-祁门高速公路、池州长江公铁两用大桥、市域轨道交通线网、秋浦河航道整治等项目前期工作。

　　完善城市功能。以海绵城市试点为抓手，重点推进平天湖、清溪河水环境整治等水生态、水安全系统建设，城市绿道、黑臭水体治理等园林绿地系统建设，秋浦西路、九华山大道综合改造工程等道路交通系统建设，学校改造工程配套海绵城市建设项目和老旧小区整治等建筑小区系统建设，努力打造全国中小城市海绵城市建设典范。

　　加强城市管理。以全国文明城市创建为抓手，大力整治群众反映强烈的市容市貌、交通秩序、背街小巷、户外广告等突出问题，选择20个老旧小区，提升物业管理，完善公共设施，综合整治环境，切实补齐短板。加快城市公交改革，建立产权清晰、运营规范、管理科学的公交运行体制机制。运行数字化城市管理平台项目，提高城市管理智能化水平。

　　培育特色城镇。全面开展新型城镇化和绿色生态城市综合试点建设，以“三治三增三提升”为抓手，进一步加强县城规划建设管理，完成县城总体规划修编和县城特色风貌等专项规划编制，深入实施“六项行动”，推动县城提质扩容，加快特色发展。力争创建省级宜居小镇、特色景观旅游名镇名村8个以上。

　　建设美好乡村。完成第三批省级中心村建设任务，开展第四批省级中心村建设，同步在25个乡镇政府驻地建成区开展环境整治，抓好青阳县美好乡村建设整县推进试点。开展农村生活垃圾专项治理三年行动，健全垃圾处理和保洁服务长效机制。完成35座小型水库除险加固和覆盖15万人的农村饮用水安全提质增效工程，实施小型水利工程改造提升和3处中小河流治理工程，完成水利投资4亿元；实施农村公路畅通工程及村级公路网化工程100公里以上、水毁及危桥加固改造36座，有序推进城乡客运一体化；实施骨干电网输变电和农村电网改造升级工程，完成电网投资8.95亿元。

　　（四）加强改革创新攻坚，切实增强发展动能

　　加快转变政府职能。加强政府权力运行监管，建立权力清单、责任清单、涉企收费清单动态调整机制，全面梳理和公开公共服务事项目录，制定政府定价项目清单。继续承接落实国家和省取消下放的行政审批事项，加快行业协会商会与行政机关脱钩，清理规范行政审批中介服务项目。探索县级部分管理权限委托下放乡镇行使，优化公共服务流程。大力推行综合执法，支持司法体制改革，依法打击违法犯罪行为，维护公平竞争的市场秩序。稳步推进事业单位分类改革，巩固公务用车制度改革成果，完成机关事业单位养老保险、县以下机关公务员职务与职级并行、工资制度改革和主城区环卫管理体制市场化改革。

　　加强国有资产资源资金管理。强化市直行政事业单位国有资产运营管理，深化财政预决算公开、预算编制执行、政府债务管理和盘活财政存量资金改革。抓好国家电子招标投标交易平台试点，推进公共资源交易市县一体化改革。巩固国有资金投资建筑工程项目专项治理成果，完善政府性投资项目管理制度，推进建设模式改革。创新投融资机制，扩大与社会资本合作范围。

　　强力招商引资。突出“两高一首”产业，梳理产业链上下游关键环节，加强与央企、知名民企、上市公司、行业龙头企业的对接合作，力争引进一批龙头项目、基地项目、创新项目。大力开展先进制造、文化旅游、健康养老养生、新兴服务业态、生态农业、基础设施等领域全方位招商，积极承接引进研发团队、营销中心、结算中心等总部经济。狠抓现有企业存量招商，支持引导20家以上企业开展资产重组、二次招商。力争引进亿元以上项目120个，实际到位资金350亿元以上，实际利用外资3.6亿美元以上。

　　激发市场主体活力。深化“三证合一”、“先照后证”、登记注册全程电子化管理等商事制度改革，全年净增私营企业1600户、个体工商户5600户以上，民营经济增加值比重提高0.5个百分点。集成扶持政策和服务资源，加快青年创业园、小微企业创业基地等孵化基地建设，积极打造众创空间、创客之家等创新创业平台，大力扶持网络创业，鼓励科研人员自主创业，支持农民工返乡创业。

　　（五）坚持生态文明先行先试，增创特色发展优势

　　筑牢生态安全屏障。编制市县（区）主体功能区规划，强化生态红线管控。继续推进国家环保模范城市、全国低碳城市、市开发区国家低碳工业园区创建，编制升金湖保护与开发规划，加快升金湖湿地生态效益补偿试点，加强城市湿地森林公园、平天湖国家湿地公园建设，严格保护越冬候鸟栖息地。抓好“千万亩森林增长工程”建设，推进“三线三边”绿化改造提升，人工造林2.3万亩，创建省级森林村庄27个、森林长廊示范段12公里，新建和改造提升城镇园林绿地25万平方米。完成长江岸线崩岸治理，实施小流域水土保持综合治理、坡耕地水土流失综合治理试点，治理水土流失20平方公里。建成主城区备用饮用水源。

　　加大环境治理力度。强化大气污染防治，开展东至经济开发区废气深度治理和挥发性有机物专项整治，巩固科技治超成果，推进齐石路矿石运输廊道建设项目招商，加强秸秆禁烧、建设施工扬尘、重点企业及矿山粉尘治理，完成淘汰黄标车年度任务，规范主城区烟花爆竹燃放行为。实施水污染防治行动计划，深化畜禽水产养殖、城镇污水、工业污水等重点污染源治理，确保污水治理设施规范运行。强化城市生活垃圾无害化处理，抓好土壤污染防治、矿山生态整治、河道采砂整顿和地质灾害防治，整顿关闭非煤矿山11家。加强环境执法和网格化监管。

　　强化资源节约集约循环利用。巩固土地督察问题整改成果，完成城市（镇）周边和全域永久基本农田划定工作，健全土地节约集约利用长效机制，依法加强土地管理，确保前5年供地率70%以上，单位生产总值建设用地消耗下降2.5%。加快水生态文明城市建设，实施最严格水资源管理制度，万元工业增加值用水量下降9%。严格环境准入和污染物总量排放控制，所有落户企业产品单耗和污染物排放必须达标，引导企业通过技术改造、上下游配套，加快水、气、固废等循环利用，大力发展循环经济。

（六）补齐公共服务短板，着力保障改善民生

　　认真实施民生工程和精准扶贫工作。坚持保基本、兜底线，实施省33项民生工程，办好市10件惠民实事。落实县区扶贫主体责任和市县定点帮扶制度，巩固精准识别建档立卡成果，动态核实贫困村、贫困户基本信息数据，编制扶贫开发“四项清单”，实施“一村一策、一户一法”，抓好年度扶贫项目建设，着力发展贫困村集体经济，确保减少贫困人口1.96万人以上。

　　加强就业和社会保障工作。完善促进就业、鼓励创业、自主择业的政策体系和服务体系，推进“工学一体”就业就学试点，创业就业和新型职业农民培训9700人，城镇新增就业1.37万人，农村劳动力转移就业1.8万人。维护劳动者合法权益。做好城镇职工基本养老保险关系转移接续，实施城乡居民大病保险制度，加强城乡低保信息化动态管理，提高农村五保、优抚对象保障水平，做好医疗救助、孤儿救助、临时救助、灾害救助工作，开展社会救助工作绩效评估。发展老龄事业，落实农村留守儿童、妇女、老年人关爱政策，保障妇女、儿童和残疾人合法权益。巩固国有房产清理成果，加强后续监管、分类处置，推进棚户区改造货币化安置，提高保障性住房分配入住率。

　　增加基本公共服务供给。实施第二期学前教育三年行动计划，完成60所义务教育阶段薄弱学校办学条件改善项目，支持高等院校、职业学校产教融合、工学结合、校企合作，扶持和规范民办教育，支持老年教育。深化城市公立医院改革，加强基层公共卫生机构能力建设，依托市人民医院组建城市医联体，选择贵池区开展县域医联体试点，力争市儿童医院主体工程完工，30%以上乡镇中心卫生院达到二级综合医院标准。巩固血防成果，抓好重大传染病、职业病防控。落实全面两孩政策。大力实施文化惠民工程，做好华龙洞、七星墩古人类遗址保护工作，力争创建国家级、省级传统村落5个。广泛开展全民健身，扩大体育消费。加强统计规范化建设，夯实统计基层基础，认真做好第三次全国农业普查工作。加强外事、侨务、对台、供销、气象、保密、档案、地方志、防震减灾、广播电视、新闻出版、社科研究等各项工作。

　　（七）加强和创新社会治理，维护社会和谐稳定

　　着力夯实社会治理基础。巩固主城区标准化社区创建成果，加快社会服务管理信息化平台建设，选择30%社区开展社区、社会组织、社工“三社联动”社会治理机制试点，计划用3年时间实现试点全覆盖。扩大农村社区建设试点，推进村务公开民主管理。大力培育和践行社会主义核心价值观，弘扬“最美”道德风尚，提升市民文明素质。充分发挥工会、共青团、妇联等人民团体的重要作用，支持关心下一代工作和红十字、慈善公益事业，大力倡导志愿服务，培育基层服务等社会组织，推进城市共治共管、共建共享。

　　加强公共安全体系建设。启动“七五”普法，加强公共法律服务体系建设，完善人民调解、行政调解、行政复议、民商仲裁等多元化纠纷解决机制。更加注重运用法治方式预防和化解社会矛盾，解决群众合理合法诉求。完善社会治安立体化防控体系，严厉打击各类违法犯罪活动。坚持实施重大决策社会稳定风险评估，健全网络舆论正确引导和突发事件应急处置机制。深入开展安全生产“铸安”行动，严防重大生产安全事故发生。争创全省首批食品药品安全城市，继续加强产品质量、市场物价、建设工程质量监管。整合资源，启动自然灾害监测预警、应急管理等统一指挥系统建设。依法维护民族团结和宗教和谐稳定。支持驻池部队建设，加强国家安全、国防教育、国防动员、民兵预备役和人民防空工作，推进军民融合发展，争创全国双拥模范城“四连冠”。

　　（八）强化政府自身建设，打造忠诚干净担当施政团队

　　保持奋发有为的进取状态。按照“三严三实”的要求，始终牢记肩负的职责和使命，坚持发展第一要务、创新第一动力，夙夜在公，扎实苦干，健全督查激励问责机制，促进各方面奋发有为、干事创业。加强学习思考和实践历练，不断研究新情况、解决新问题，摆脱传统增长的路径依赖，提高引领新常态、推动新发展的专业化水平。

　　提升依法行政的能力水平。坚持法无授权不可为、法定职责必须为，自觉运用法治思维和法治方式，健全重大事项决策规则和程序，严格按照法定权限和程序行使权力、履行职责。自觉接受人大的法律监督和工作监督，主动接受政协的民主监督和社会各界的监督，充分听取和吸纳各民主党派、工商联、无党派人士及各人民团体意见。加强政务公开和政府信息公开，让权力在阳光下运行。

　　强化服务发展的责任担当。始终践行群众路线，多做打基础、利长远、惠民生的实事好事，一环紧着一环拧，一锤接着一锤敲，切实把好的蓝图绘到底。坚持敢于担当，狠抓落实，对重点工作紧抓不放，加强协调调度，严格督查督办，强化绩效考核，大力褒先惩后。坚决整治“庸懒散拖”，认真解决“门好进、脸好看，事还是难办”的问题，不断提高行政效率。

　　严格廉洁从政的制度落实。认真落实中央八项规定和省委、市委各项规定，模范遵守廉洁自律准则，严防“四风”反弹，自觉廉洁从政、廉洁用权、廉洁修身、廉洁齐家。严格落实党风廉政建设主体责任，深入推进政府系统廉政建设，严肃财经纪律，严控“三公”经费，加强行政监察、审计监督和执纪问责，今年重点推进市直系统事业单位内审全覆盖。各级政府公务人员都要严格要求自己，勤勉尽责、秉公用权，抓好班子、带好队伍，涵养正气、弘扬清风，进一步树立为民务实清廉政府形象。

　　各位代表！全面小康前景无限美好，梦想成真需要同心创造。让我们紧密团结在以习近平同志为总书记的党中央周围，在省委、省政府和市委的坚强领导下，凝聚全市人民的智慧和力量，开拓创新，真抓实干，奋力开创全面建成小康社会决胜阶段新局面，为加快“三区”发展、建设幸福池州而努力奋斗！
